# Personal Care Aides as Household Employees and Independent Contractors: Estimating the Size and Job Characteristics of the Workforce

**DOI:** 10.1093/geroni/igab049

**Published:** 2021-11-29

**Authors:** Joy Jeounghee Kim

**Affiliations:** School of Social Work, Rutgers, The State University of New Jersey, New Brunswick, New Jersey, USA

**Keywords:** Fair Labor Standards Act (FLSA), Home- and community-based services (HCBS), Long-term services and supports, Self-directed Medicaid

## Abstract

**Background and Objectives:**

Although studies pointed out that the number of personal care aides (PCAs) at risk of being in informal employment arrangements is sizeable, little is known about its size and worker characteristics. This study aimed to estimate the share of PCAs working as household employees or independent contractors. It also aimed to compare their basic job characteristics against the job characteristics of those working as agency and government employees.

**Research Design and Methods:**

Using data from the 2014–2018 American Community Surveys, a sample of 43,287 PCAs working for pay in the home- and community-based service (HCBS) industry was identified, and their job characteristics—full-time weekly work (i.e., working at least 35 hours per week), year-round work (i.e., working at least 50 weeks a year), and annual gross earning—were analyzed by their employment arrangement.

**Results:**

Analyses found that (a) close to a quarter of aides in the HCBS industry work as household employees or independent contractors while their share in the workforce varies by state and that (b) the work hours and earnings of full-time year-round working household employees or independent contractors are *greater* than those of their agency counterparts. The results shed light on why some aides may work as household employees or independent contractors.

**Discussion and Implications:**

The presence of household employees and independent contractors has important implications for PCAs’ job characteristics and labor shortage in the U.S. home care industry. Considering the potentially negative consequences for both the aides’ economic security and the quality of care that consumers can receive, attention should be paid to ways to bring the aides into a more formal employment arrangement.

**Translational Significance:** The study examined personal care aides’ job characteristics by their employment arrangement using a nationally representative sample of aides drawn from the American Community Surveys. The study found that nearly a quarter of personal care aides worked as household employees and independent contractors and their earnings were greater than the earnings of agency-employed aids when working full-time year-round. The results have important implications for personal care aids’ job characteristics and worker shortage in the home care industry.

## Background

As baby boomers become older, the demand for long-term services and supports (LTSS) has been on the rise in the United States. While much of LTSS needs are met by unpaid families and friends, many individuals also rely on paid caregivers. Efforts to promote independence and community integration of the LTSS populations drove the services to be home- and community-based services (HCBS) (Stone, 2010). One of the most crucial elements of HCBS is home care services designed to assist individuals with daily living and instrumental activities (e.g., bathing, dressing, toileting, cleaning, cooking, etc.) at their residence. Approximately 28% of individuals with LTSS needs rely solely on public programs such as Medicaid, Medicare, Workers’ Compensation, and Veterans’ programs for their home care services in the United States (Janus & Ermisch, 2015). Most of those who are ineligible for the public programs, however, use personal funds to pay for the services. At least 65% of home care services for all income groups are paid for in whole or in part by personal funds, and the percent paid solely by personal funds goes up to 97% for individuals with annual family incomes of at least $75,000 (Janus & Ermisch, 2015). For those relying on personal funds, the affordability of home care services is crucial to maintaining their independence at home and in communities.

The workforce that provides home care services is 2 million personal care aides (PCAs) who help LTSS populations across the life span with daily living and instrumental activities in diverse settings (i.e., individual and family services; residential, developmental, or mental health facilities; home health care services; and continuing care, assisted living, and retirement communities; U.S. Bureau of Labor Statistics, 2018). The aides earn, on average, $11.59 per hour and $24,100 annually and are one of the lowest-paid workers in the United States (U.S. Bureau of Labor Statistics, 2018). Not only is their hourly wage low, but they receive few employment benefits (Dawson, 2016). As most individuals with LTSS needs do not require full-time care, many PCAs have only part-time work hours (Dawson, 2016). Furthermore, PCAs in HCBS used to be excluded from the minimum wage and overtime rules of the Fair Labor Standards Act (FLSA) for decades, for being domestic workers outside interstate commerce and for being casual companion providers. Although the recently revised FLSA rules extended the law’s coverage to third-party hired PCAs starting in January 2016 (Dawson, 2016), anecdotal evidence suggests that many agency employers introduced new scheduling practices to avoid overtime payment and made PCAs piece together work hours with multiple employers but still without overtime compensations (Iezzoni et al., 2019).

PCAs are hired in a variety of ways in the United States. Home care beneficiaries of public programs typically hire a PCA through a Medicaid-/Medicare-participating home care agency, and the beneficiaries of self-directed Medicaid programs can hire a PCA directly or through a registry, depending on their state program. A privately paying consumer, on the other hand, can hire a home care agency or directly hire a PCA as a *household employee or an independent contractor*. In these informal arrangements, PCAs may run the risk of being *unreported workers*, whose employers fail to pay employment taxes on their behalf. Although informal employment arrangements are efforts to satisfy individual needs and preferences while containing the costs of care, they can have important implications for the growth and quality of the home care workforce. Unlike the agency employees who have some training requirements in 29 states and the District of Columbia (Paraprofessional Healthcare Institute, 2018), most states do not impose occupational training or certification requirements for PCAs working as independent contractors or household employees. The share of independent contractors and household employees, therefore, has implications for the quality of the overall workforce (Newquist et al., 2015). Furthermore, a lack of oversight, support, and protection for either consumers or PCAs leaves both parties vulnerable to a range of potential personal and professional risks (Iezzoni et al., 2019; Newquist et al., 2015). These arrangements also have implications for the supply of the workforce. Some agencies feel that the presence of informal arrangements exacerbates worker shortages as agencies find themselves competing with private households to hire PCAs (Doty, 2017).

Unfortunately, knowledge of privately paid home care services, let alone PCAs in informal arrangements, is scarce, representing a large gap in the literature (Newquist et al., 2015). Although numerous studies pointed out that the so-called gray market that involves unreported workers is sizeable in the home care industry in the United States, little is known about its size and worker characteristics (Dawson, 2016; Howes, 2014; Schierholz, 2014; Smith, 2008; U.S. Government Accountability Office, 2017). Without knowing the size of this group, however, it is difficult to estimate its impacts and debate its implications. While it would be impossible to estimate the size of unreported PCAs, data from the American Community Survey (ACS) make it possible to examine those who are directly hired by private households with the risk of being unreported workers. In what follows, I review the motivations of both PCAs and customers to enter into a direct employment relationship and discuss its implications for PCAs’ job characteristics. Using ACS data, I estimate the share of PCAs by their employment arrangement (i.e., the type of employer) and also compare their basic job characteristics by employment arrangement. I conclude with the implications of PCAs’ employment arrangement for the workforce supply and the cost and quality of home care services.

## PCA’s Employment Arrangement in the United States

### Household Employees and Independent Contractors

PCAs in the United States work as independent contractors or as the household employees of consumers in part because the workers and their consumers want to avoid home care agencies’ control and overheads. PCAs, for example, may be willing to work as household employees or independent contractors when they want to work longer hours than a home care agency may allow, or want to work for consumers willing to pay more than the prevailing agency rates (Dawson, 2016). At the same time, consumers can be motivated to directly hire PCAs to save about 20%–30% of the fees that a home care or employment agency typically charges. They may also want to maximize their choices in hiring PCAs sometimes by hiring the aide of their choice, not the worker an agency chooses. It is also possible that they want to circumvent state and agency rules on the tasks PCAs can perform or want to ask PCAs to offer a range of support services broader than an agency typically allows.

When a consumer hires a PCA outside an agency, they recruit by personal referral or word of mouth (from families, friends, or neighbors) or using caregiver registries that list potential caregivers already screened and vetted. In the United States, a consumer who hires and supervises a domestic worker and pays at least $2,300 annually is a *household employer* and is responsible for complying with the rules set by Internal Revenue Services (IRS) and the FLSA (Internal Revenue Services, 2020). That is, the customer is required to pay payroll, unemployment, and workers’ compensation taxes and abide by the prevailing minimum wage and overtime pay rules. The customer can carry out such responsibilities on his/her own or receive assistance from many registries that offer administrative and payroll services.

Beneficiaries of Medicaid self-directed programs in the United States can also become household employers of their PCAs, often called independent providers. With greater control of services and service delivery, the beneficiaries are allowed to select and hire an independent provider of their choice, including their relatives or friends, or through public caregiver registries (Centers for Medicare & Medicaid Services, 2008; Scales, 2019). Yet, control over the independent providers’ hourly wage rates and benefits often rests on state or county authorities or agencies unless the beneficiaries receive a monthly budget to purchase a range of goods and services to meet their needs. This suggests that various employers’ roles relevant to IRS and FLSA rules are dispersed among multiple parties including fiscal intermediary or public or quasi-public entities. Medicaid beneficiaries can elect to use all or any part of the services offered by the entities to become private household employers either solely or jointly with the entities (Flanagan & Green, 1997; U.S. Department of Health and Human Services, 2019; U.S. Government Accountability Office, 2017). This arrangement is seen in some states of the United States such as Michigan, Massachusetts, Oregon, New York, and California. The fragmented employment roles and responsibilities bring up ambiguity and challenges regarding employer liability (Howes, 2014; Smith, 2008).

Despite the mandated employer responsibilities, a sizeable number of consumers who directly hire PCAs with private funds do not fulfill these responsibilities, and sometimes misclassify their PCAs as *independent contractors* (Christman & Connolly, 2017). The share of PCAs working as independent contractors can be considerably large among those paid with private funds given a large number of consumers who are ineligible for public services or cannot afford (or choose not) to pay for agency services (U.S. Department of Health and Human Services, 2019). Furthermore, PCAs paid with *public funds* can also be classified as independent contractors. It is because the aforementioned independent providers under some Medicaid self-directed programs can be treated as independent contractors when states do not take on the role of “employer of record” and contract with companies for fiscal intermediary services. Although the federal government provides guidelines against such a practice for Medicaid self-directed programs, it is unknown to what extent independent providers in those programs can avoid being classified as independent contractors (U.S. Department of Health and Human Services, 2019).

### State Variation in PCAs’ Employment Arrangement

Employment arrangement defines if a PCA is an employee or an independent contractor, and who employs them if they are an employee. The share of PCAs in a certain employment arrangement is expected to vary by state. It is because the factors that affect the demands for home care—the demographic characteristics of their long-term care populations, availability of publicly funded programs, the supply of home care agencies, and presence of alternative sources of care—vary by state (Ng et al., 2015). California, Texas, and New York—the three states that hire as many as 40% of the nation’s PCAs—manifest the variation in some of these factors relevant to employment arrangements. For instance, these states have various per capita expenditures on Medicaid HCBS that fund home care for low-income individuals with LTSS needs. In 2013, Texas with 12% of its population over 65 years old spent less than $10,000 per capita, and California spent between $10,000 and $20,000 per capita with 13.6% of the population in the 65 years or older group. New York had more than $30,000 per capita expenditure with 15% of its population in the older adult group (Kahn et al., 2015; Rolf, 2016). States with more per capita spending on HCBS and greater availability of publicly funded home care services may have a lower share of PCAs working as household employees or independent contractors. Some of the funds could be spent on supporting and training PCAs to better integrate them into the health care system (e.g., California Long-Term Care Education Center, 2016).

Other state variations in the home care industry, such as the presence of unions and worker cooperatives, can also create variations in PCA’s employment arrangements as unions and worker cooperatives can affect PCAs’ wages and benefits. California, with its largest number of PCAs in its In-Home Support and Services, established a county-based Public Authority that serves as the employer of record for PCAs (Dawson, 2016). Therefore, PCAs in California can be employees of counties’ Public Authority, unionized, and bargain with the Authority for their working conditions and wages (Dawson, 2016). Similarly, in New York where the country’s largest home care worker cooperative exists, PCAs can be the cooperative’s employees, benefit from better wages, and access to union membership and job training (Kennelly & Odekon, 2016). With this background, below I estimate the percentages of PCAs working as household employees and independent contractors, examine state variations in the percentages, and compare PCA’s basic job characteristics by employment arrangement.

## Method

### Data and Sample

Data for this study came from the 2014–2018 ACS. ACS is an ongoing national survey of a sample of about 3.5 million addresses conducted by the U.S. Census Bureau to collect social, economic, housing, and demographic information of the U.S. population (Ruggles et al., 2020). The survey is collected nearly every day of the year, and data are pooled across a calendar year to generate estimates of the year (Ruggles et al., 2020). ACS is ideal for this study as it has a large sample size for PCAs and information on their employment characteristics. Using the occupation code that reports the survey respondent’s primary occupation, a total sample of 43,287 PCAs working for pay in the HCBS industry across the 5 years was identified (*N* = 8,140 for 2015, *N* = 8,540 for 2016, *N* = 8,808 for 2017, *N* = 9,138 for 2018, *N* = 8,661 for 2019). The industry codes were used to identify PCAs working in three HCBS industries: individual and family services, home health care services, and private household industries. The PCAs who did not work or had zero earnings were excluded from the sample. The sample also excluded the owners of an incorporated business because the focus of this study was to examine PCAs’ employment relationships with their employers, and those who own incorporated businesses in the home care industry are *not* likely to be employees or independent contractors (i.e., sole proprietors; Connolly, 2015).

There are a couple of notable limitations in ACS data. The first limitation is that PCAs who combine multiple types of work—including work offered by private households and agencies—cannot be identified because the survey captures the primary source of work for each respondent. What this means is that ACS data may *not* include many independent providers in Medicaid self-directed programs, whose PCA job is a secondary source of income. The second limitation of ACS data is that it is unclear how PCAs with joint employers are recorded in the data. Some states’ self-directed Medicaid programs allow joint employers between household employers and public employers (e.g., Oregon). However, how ACS participants answered the survey question on employer classification is unknown. The third limitation of ACS is that it is uncertain how well the survey captures unreported workers and undocumented immigrants, who may work as PCAs. These limitations of ACS indicate that the result of this study is likely to be an *underestimate* of the share of PCAs working as household employees and independent contractors in the HCBS industry. It is important to note that underestimation can be substantial in case of employee misclassification, which was reported as prevalent practice in the home care industry (Connolly, 2015). Despite these limitations, however, ACS data remain the source of the closest systematic estimation of privately hired PCAs in the United States (Burnham & Theodore, 2012).

### Variable Measures and Data Analyses

The key variable of interest for this study is five categories of employment arrangement, measured with a combination of the *class of worker* (employees of private, nonprofit, or government agencies or self-employed) and *industry* variables in ACS data. Employment arrangements were measured if a PCA was (a) a private agency employee, (b) a nonprivate agency employee (i.e., state- or county-run home care agencies), or (c) a government employee (i.e., an employee of federal, state, or local government). PCAs who worked in the private household industry as private wage earners were classified as (d) household employees. When a PCA worked as a self-employee in one of the three HCBS industries, they were classified as (e) an independent contractor (note that incorporated business owners were excluded from this study).

PCAs’ job characteristics are measured with full-time year-round work status and annual earnings. Full-time and year-round work refers to working 35 hours or more per week and 50 weeks or more year-round. Annual earnings refer to annual gross earnings adjusted to the year 2019 for inflation. These variables are used as basic job characteristic indicators for PCAs because securing enough work hours and achieving earnings stability are reported to be the primary concerns for many PCAs (Osterman, 2017, p. 34).

In terms of data analyses, I first estimated the weighted percentages of PCAs working as household employees and independent contractors in all states, the selected three states (California, New York, and Texas), and each of the rest of states other than the three states (referred as “Other” states). I then examined if and how the employment characteristics of household employees and independent contractors differed from those of PCAs in other employment arrangements. Using the most common Fisher’s Least Significant Difference pairwise multiple comparisons in SAS GLM procedure (SAS, 2018, p. 4004), I examined if the mean earnings of household employees and independent contractors statistically differed from the mean earnings of PCAs in other employment arrangements. Because the distribution of earnings data is generally skewed by outliers, I also conducted two-sample median tests using SAS NPAR1WAY procedures (SAS, 2015, p. 6652) to assess whether or not the median earnings of household employees and independent contractors statistically differed from the median earnings of PCAs in other employment arrangements. In addition, using Fisher’s Least Significant Difference pairwise multiple comparison tests in SAS GLM procedure, I examined if and how the rates (expressed as percentages here) of full-time year-round working PCAs among household employees and independent contractors differed from the rates (expressed as percentages here) of those in other employment arrangements.

Particular attention was paid to the employment arrangement of aides who lived in the three states—California, Texas, and New York—that had nearly 40% of all PCAs in the United States. Because of sufficient PCAs in each state, these three states allowed one to compare state variations in the share of household employees and independent contractors. I estimated the mean and median earnings of full-time year-round working PCAs by employment arrangements and assessed if the earnings were higher among household employees and independent contractors than the earnings of PCAs in other employment arrangements. This was done to isolate the earnings of full-time year-round workers from the earnings of others who may work part-time part-year voluntarily for work–family balance or work schedule flexibility.

## Findings

### The Percentages of Household Employees and Independent Contractors

As the first column of Table 1 shows, about 22.00% of the sample PCAs were either household employees (9.37%) or independent contractors (12.50%). A little more than a half (53.20%) of PCAs were private agency employees, and about a quarter were the employees of nonprivate or government agencies (10.00% and 14.93%, respectively). Looking at the select three states, the percentage of independent contractors varied by state. Nearly 12.00% of PCAs in California were independent contractors, but the percentage was only about 7.00% in New York. The share of household employees varied less ranging from 6.10% in Texas and 8.91% in California. As suggested above, California had more than 40.00% of PCAs classified as government employees, significantly higher than in Texas (4.72%) and New York (6.78%). New York, on the other hand, had a notably higher percentage of PCAs employed by nonprivate agencies (17.50%), compared to less than 4.00% in Texas and 5.00% in California. The highest percentages of PCAs in Texas (75.28%) and New York (61.48%) were employees of private agencies, but only 33.98% in California were employed by private agencies. In “Other” states, a higher percentage of PCAs worked as household employees (10.29%) or independent contractors (13.70%), indicating that nearly a quarter of the aides were in the employment arrangements that may expose them to the risk of being informal workers. Please note that the Supplementary Table presents large state-by-state variations in the percentage distributions of PCAs’ employment arrangements. The table shows that in “Other” states, Florida and Minnesota were the states with the highest (34.66%) and lowest (3.72%) percentages of PCAs working as independent contractors. In terms of the percentages of PCAs working as household employees, Connecticut (21.06%) and North Dakota (3.72%) were the states with the highest and lowest percentages, respectively.

**Table 1.** Weighted Percentage Distribution of Employment Arrangement of Personal Care Aides in the Home- and Community-Based Service Industry

State	Private agency employee	Nonprivate agency employee	Government employee	Household employee	Independent contractor
All states	53.20	10.00	14.93	9.37	12.50
California	33.98	4.96	40.45	8.91	11.71
New York	61.48	17.50	6.78	7.07	7.16
Texas	75.28	3.73	4.72	6.10	10.17
Other states^a^	56.25	12.10	7.66	10.29	13.70

*Note*:

^a^Detailed percentage distribution of employment arrangement in each of these states is presented in the Supplementary Table.

### Job Characteristics of Household Employees

Data analyses revealed that the mean and median annual earnings of all PCAs were $17,650 and $13,910 and approximately 38.60% of PCAs worked full-time year-round. Table 2 presents findings from statistical significance testing of the job characteristics of household employees, compared to job characteristics of PCAs’ in other employment arrangements. While the table presents many statistics, the overall findings suggest that household employees had higher or highest earnings of all PCAs in other employment arrangements, and the percentage of them working full-time year-round was compatible with the percentage of those in other employment arrangements.

**Table 2.** Comparisons of Personal Care Aides’ (PCAs’) Job Characteristics by Employment Arrangement, Weighted

Job characteristics	Household employee	Independent contractor	Private agency employee	Nonprivate agency employee	Government employee
Mean annual earning ($) of all PCAs^a^					
All states	19,155_(b)_	18,204_(a)_	16,911_(a),(b)_	19,920_(b)_	17,357_(a)_
California	20,704	19,487	19,026_(a)_	18,822	16,672_(a),(b)_
New York	21,824_(b)_	16,566_(a)_	18,670_(a)_	21,188_(b)_	22,347_(b)_
Texas	17,287	15,596	13,470_(a),(b)_	13,815	15,853
Other states	18,627	18,174	16,935_(a),(b)_	20,191_(a),(b)_	18,417
Median annual earning ($) of all PCAs^b^					
All states	14,280_(b)_	12,240_(a)_	13,910_(b)_	17,680_(a),(b)_	12,840_(a),(b)_
California	16,800_(b)_	14,352_(a)_	15,600_(b)_	15,808	12,648_(a),(b)_
New York	15,408_(b)_	12,190_(a)_	16,000_(b)_	19,000_(a),(b)_	20,400_(a),(b)_
Texas	13,668	10,700	10,700_(a)_	11,220	10,302_(a)_
Other states	13,589_(b)_	12,000_(a)_	14,000_(a),(b)_	18,000_(a),(b)_	14,000_(a),(b)_
Percentage (%) working FTYR^a^					
All states	39.27_(b)_	33.81_(a)_	38.70_(b)_	47.05_(a),(b)_	35.88_(a),(b)_
California	43.96_(b)_	33.53_(a)_	41.72_(b)_	42.43_(b)_	33.73_(a)_
New York	40.36	32.75	44.80	52.19_(a),(b)_	54.46_(a),(b)_
Texas	45.05	42.83	31.26_(a),(b)_	40.98	27.03_(a),(b)_
Other states	37.12_(b)_	32.92_(a)_	38.85_(b)_	47.30_(a),(b)_	39.31_(b)_
Mean annual earning ($) of PCAs working FTYR^a^					
All states	31,644	30,359	26,131_(a),(b)_	28,011_(a),(b)_	27,630_(a),(b)_
California	32,266	28,928	28,431_(a)_	28,619	27,084_(a)_
New York	37,984_(b)_	26,525_(a)_	26,603_(a)_	28,701_(a)_	31,546_(a)_
Texas	25,385	22,534	21,877_(a)_	22,837	24,291
Other states	31,656	32,195	26,207_(a),(b)_	28,025_(a),(b)_	28,294_(a),(b)_
Median annual earning ($) of PCAs working FTYR^b^					
All states	25,440_(b)_	21,632_(a)_	22,577_(a)_	24,960_(b)_	23,816_(a),(b)_
California	26,750_(b)_	22,000_(a)_	25,000_(a)_	24,960	23,562_(a)_
New York	30,000_(b)_	22,500_(a)_	23,532_(a)_	26,000_(a),(b)_	31,800_(b)_
Texas	22,880_(b)_	18,720_(a)_	19,080_(a)_	21,216_(b)_	21,424_(a)_
Other states	24,960_(b)_	22,000_(a)_	22,470_(a)_	24,960_(b)_	23,816

*Notes*: FTYR = full-time year-round. “(a)” represents that the characteristic was significantly different from that of *household employees* at *p* < .05; “(b)” represents that the characteristic was significantly different from that of *independent contractors* at *p* < .05.

^a^Statistical significance of the differences in these means and rates (expressed as percentages) was tested with Fisher’s Least Significant Difference pairwise multiple comparisons.

^b^Statistical significance of the differences in these medians was tested with two-sample median tests.

Table 2 first presents the results of pairwise multiple comparisons of the PCAs’ mean earnings focusing on how the earnings of household employees differed from those of PCAs in other employment arrangements. It is worthwhile to point out that the annual mean earnings of household employees were *greater* than the earnings of private agency employees in all states ($19,155 vs $16,911), California ($20,704 vs $19,026), New York ($21,824 vs $18,670), Texas ($17,287 vs $13,470), and “Other” states ($18,627 vs $16,935), as well as the earnings of government employees in all states and California. In California and Texas, household employees had the highest mean earnings of all types of employees.

The results of median tests of the earnings reveal that the median earning of household employees was not statistically different from those of private agency employees but the second-highest in all states, followed by the earnings of nonprivate agency employees. In California and Texas, household employees had the highest median earnings compared to those in other employment arrangements. Californian household employees had the median earnings of $16,800, significantly higher than those of government employees ($12,648) and independent contractors ($14,352). In “Other” states, however, household employees’ median earning was significantly lower than nonprivate agency employees’ median earning ($13,589 vs $18,000).

Although the percentages of PCAs working full-time year-round varied by their employment arrangement, most of them did not work full-time year-round. Pairwise multiple comparison tests indicated that the percentage working full-time year-round for household employees (39.27%) was not statistically different from the percentage for private agency employees (38.70%) in all states, but higher than the percentages for government employees (35.88%) and independent contractors (33.81%). Nonprivate agency employees had the highest rate of working full-time year-round in all states. A similar pattern of working full-time year-round was observed in “Other” states. In both California and Texas, household employees had the highest percentages of PCAs working full-time year-round at 43.96% and 45.05%, respectively.

The bottom rows of Table 2 show the annual earnings of full-time year-round working PCAs to set them apart from the earnings of part-time part-year workers. Not only the mean earnings but also the median earnings of household employees were consistently greater than those of private agency employees among full-time year-round workers. For example, the mean annual earning of private agency employees was $26,131 but that of household employees was $31,644, the highest of all groups in all states. The pairwise comparisons of mean earnings underscored the finding that full-time year-round working household employees earned significantly and consistently *higher* than private agency employees in all the states under study. In addition, they earned *more* than nonprivate agency employees and government employees in most states. The median tests also revealed similar findings. In all states, for example, full-time year-round working household employees had the highest median earnings ($25,440), compared to the earnings of private agency employees ($22,577), government employees ($23,816), and independent contractors ($21,632).

### Job Characteristics of Independent Contractors

Table 2 also presents findings from statistical significance testing of the job characteristics of independent contractors, compared to the job characteristics of PCAs’ in other employment arrangements. The mean earnings of independent contractors in all states were either significantly *higher than or similar to* the earnings of private agency employees. They were also greater than the earnings of government employees in California. New York was a place where PCAs working as independent contractors had significantly *less* average earnings than those working as employees. Independent contractors in all states, however, had the *lowest median* earnings ($12,240), compared to nonprivate agency employees ($17,680), private agency employees ($13,910), and government employees ($12,840). Regardless of the states under study, the median earnings of independent contractors were either significantly *lower than or similar to* the median earnings of private agency employees. The discrepancies in the mean and median earnings suggest that there were some independent contractors whose earnings were considerably higher than the earnings of private agency employees.

About full-time year-round work, pairwise multiple comparison test results show that a *lower* percentage of independent contractors (33.81%) worked full-time year-round than those in other employment arrangements, in general. Texas, however, was a state where independent contractors (42.83%) had a *higher* percentage working full-time year-round compared to private agency (31.26%) and government employees (27.03%).

Among full-time year-round workers, the mean annual earnings of independent contractors were *the highest* at $30,359, along with household employees who earned $31,644. In fact, in “Other” states, independent contractors had the highest mean annual earning of all types of employees. In California, New York, and Texas, the mean earnings of independent contractors were not statistically different from those of any type of employee. The median annual earnings of independent contractors, however, were *the lowest* compared to PCAs in other employment arrangements. Again, these discrepancies in the mean and median earnings of independent contractors indicated the presence of some independent contractors with substantially higher annual earnings than those in other employment arrangements.

## Implications

The results of this analysis showed that close to a quarter of the personal care workforce in the HCBS industry of the United States worked as household employees or independent contractors. As indicated above, this is likely to be an underestimation because the ACS data may have undercounted household employees and independent contractors due to underreporting and employee misclassification. Although the extent of underestimation is difficult to gauge, it is important to note that the impact of underestimation may not be negligible. Comparisons of the job characteristics of household employees and independent contractors to the job characteristics of other employees suggest that the potentials for higher earnings, particularly when they work full-time year-round, may be one of the reasons why some PCAs work as household employees or independent contractors.

Interestingly, PCAs working as household employees do not seem to have more limited access to full-time year-round work. It is important to highlight that household employees generally had higher mean annual earnings than the earnings of employees of private, nonprivate, and government agencies. When they worked full-time year-round, their mean annual earnings typically surpassed $30,000, and in some places like New York, the mean earning reached nearly $38,000, more than $10,000 higher than the earning of private agency employees. For full-time year-round working household employees, even their median earnings were generally the highest compared to the earnings of PCAs in other employment arrangements.

PCAs working as independent contractors, on the other hand, seem to have a lower chance of working full-time year-round than those employed by agencies. Their median annual earnings tend to be lower than those of agency employees. However, their mean annual earnings surpassed those of agency employees, indicating that some independent contractors were earning significantly more than agency employees. This seemed particularly true for full-time year-round working independent contractors in most states because their mean annual earnings were consistently higher than those of all agency employees working full-time year-round. Although these findings cannot serve as evidence of some of PCAs’ motivations to work as household employees and independent contractors, they are suggestive of *the potential* that a higher earnings possibility may lure them into those employment arrangements. Given the fact that most PCAs lack full-time year-round employment and nonwage compensations (U.S. Government Accountability Office, 2016), the potential to make higher earnings may significantly influence their decision to work as household employees or independent contractors. Yet, as discussed above, these workers may experience a higher risk of being unreported or misclassified workers as well as experiencing labor market injustice.

Besides the implications for workers’ rights to economic security and labor market protection, employment arrangements also have significant implications for the quality of care that the PCAs provide. Because many states do not require training of PCAs even for those paid by Medicaid-funded programs, PCAs employed directly by consumers or through private-pay arrangements (i.e., independent contractors or household employees) are even less likely to be trained, certified, supervised by registered nurses regularly, and required to renew their certification (Marquand & Chapman, 2014; Newquist et al., 2015; Paraprofessional Healthcare Institute, 2018). The lack of training requirements undermines the quality of the workforce, and the quality issue may be more acute for the workforce in less formal employment arrangements. The presence of a substantial size of household employees and independent contractors also has implications for the worker shortages in the more formal labor market as home care agencies see direct hires by consumers as the most serious competition (Doty, 2017). States with generous public home care programs may have a lower share of PCAs working as household employees or independent contractors. The presence of these employment arrangements for PCAs speaks to the poor job characteristics of the more formal labor market in the home care industry as well as the lack of access to publicly funded quality home care services for middle-income consumers in the United States.

Despite their significant implications, factors contributing to certain arrangements and their consequences for PCAs and consumers are still unclear. In addition to the factors related to LTSS mentioned above (e.g., availability of publicly funded home care services for middle-income consumers or alternative sources of care, presence of public authorities for self-directed home care programs and worker cooperatives, etc.), state variations in minimum wage and overtime pay rules may also affect PCAs’ employment arrangement. By setting the wage and earnings floor, these rules may affect both the supply and demand of home care services by affecting PCAs’ wages and the prices of home care services. Future studies, therefore, need to investigate state variations in these rules and their relationships with PCAs’ employment arrangements. For such studies, detailed and historical data may be needed for both publicly funded state home care programs and alternative sources of LTSS, as well as state minimum wage and overtime pay rules.

If the supply of PCAs cannot keep up with the demand for their services in the future, the prices of home care may increase, and some consumers may find the services less affordable. This may encourage more customers to directly hire PCAs to bypass agencies’ overheads. Considering the potentially negative consequences for both the aides’ job qualities and the quality of care that consumers can receive, more attention should be paid to ways to bring the aides into more formal employment arrangements that accompany policy protections and professional supervision. Many past efforts have been made to improve the job qualities of the workforce such as labor organizing, state Medicare reform, and occupational credentialing, but without significant and broad success (e.g., Appelbaum & Leana 2011; Kelly et al., 2013; Osterman, 2008, 2019). Recently, the Biden administration proposed to improve the care infrastructure as one of the American Jobs Plan priorities, and a large part of the investment is aimed to expand access to HCBS under Medicaid and to improve the job qualities of PCAs with higher wages, more benefits, and access to unions (The White House, 2021). It remains to be seen in the future how the initiative, if implemented, helps move PCAs into employment arrangements that guarantee substantially better job qualities.

## Supplementary Material

igab049_suppl_Supplementary_Material
