# Supplementary material for: Personal Care Aides as Household Employees and Independent Contractors: Estimating the Size and Job Characteristics of the Workforce
Source: Innov Aging. 2021 Nov 29;6(1):igab049. doi: 10.1093/geroni/igab049 (PMC8719739; doi:10.1093/geroni/igab049)
Supplement: igab049_suppl_Supplementary_Material [file igab049_suppl_supplementary_material.docx]

Supplemental Table: Weighted Percentage Distribution of Employment Arrangement of Personal Care Aides in the Home and Community-Based Service Industry

| State | Unweighted *N* | Private agency employee | Non-private agency employee | Government employee | Household employee | Independent contractor |
| --- | --- | --- | --- | --- | --- | --- |
| AL | 362 | 47.9 | 8.89 | 2.65 | 12.91 | 27.65 |
| AK | 105 | 52.65 | 33.76 | 3.14 | 4.61 | 5.84 |
| AZ | 1,129 | 55.16 | 12.05 | 6.48 | 12.37 | 13.94 |
| AR | 376 | 47.52 | 11.75 | 13.32 | 9.94 | 17.47 |
| CO | 553 | 61.22 | 10.21 | 5.55 | 10.41 | 12.6 |
| CT | 527 | 55.2 | 11.04 | 4.4 | 21.06 | 8.31 |
| DE | 68 | 70.22 | 9.03 | 2.25 | 8.96 | 9.54 |
| DC | 37 | 57.87 | 19.79 | 5.03 | 7.52 | 9.79 |
| FL | 1,145 | 42.01 | 4.02 | 2.29 | 17.02 | 34.66 |
| GA | 541 | 53.11 | 4.07 | 4.81 | 13.83 | 24.18 |
| HI | 118 | 43.32 | 12.59 | 8.49 | 6.58 | 29.02 |
| ID | 270 | 65.53 | 7.54 | 7.09 | 9.1 | 10.74 |
| IL | 1,797 | 57.82 | 11.58 | 11.77 | 7.17 | 11.65 |
| IN | 633 | 65.44 | 16.02 | 3.23 | 7.42 | 7.89 |
| IA | 304 | 48.27 | 25.84 | 7.15 | 7.32 | 11.43 |
| KS | 318 | 57.43 | 16.83 | 5.88 | 12.74 | 7.11 |
| KY | 404 | 45.65 | 10.91 | 6.47 | 17.7 | 19.27 |
| LA | 671 | 64.35 | 8.68 | 5.29 | 8.07 | 13.6 |
| ME | 254 | 50.88 | 25.89 | 2.81 | 9.18 | 11.23 |
| MD | 402 | 48.88 | 13.21 | 5.7 | 12.86 | 19.35 |
| MA | 957 | 51.34 | 19.26 | 6.37 | 14.05 | 8.98 |
| MI | 1,143 | 53.19 | 10.23 | 12.71 | 10.31 | 13.55 |
| MN | 871 | 70.74 | 16.23 | 5.41 | 3.89 | 3.72 |
| MS | 263 | 53.42 | 3.19 | 8.83 | 12.85 | 21.71 |
| MO | 857 | 63.38 | 15.01 | 6.75 | 6.48 | 8.38 |
| MT | 125 | 47.19 | 24.33 | 10.38 | 7.2 | 10.9 |
| NE | 171 | 46.92 | 11.81 | 9.24 | 4.79 | 27.25 |
| NV | 266 | 69.19 | 7.4 | 4.29 | 5.56 | 13.56 |
| NH | 182 | 45.33 | 30.89 | 4.63 | 10.06 | 9.1 |
| NJ | 615 | 58.51 | 7.94 | 6.73 | 15.08 | 11.74 |
| NM | 698 | 64.43 | 10 | 5.44 | 9.44 | 10.68 |
| NC | 826 | 57.55 | 7.29 | 3.79 | 12.94 | 18.43 |
| ND | 74 | 37.66 | 30.2 | 8.23 | 3.72 | 20.18 |
| OH | 997 | 63.09 | 10.33 | 5.22 | 7.14 | 14.22 |
| OK | 347 | 62.75 | 5.84 | 4.09 | 7.24 | 20.08 |
| OR | 930 | 42.4 | 11.69 | 19.87 | 11.96 | 14.07 |
| PA | 1,898 | 61.97 | 16.15 | 5.62 | 8.85 | 7.42 |
| RI | 97 | 49.08 | 30.94 | 1 | 11 | 7.98 |
| SC | 452 | 57.4 | 5.55 | 12.18 | 7.01 | 17.86 |
| SD | 71 | 43.53 | 29.45 | 11.16 | 8.43 | 7.42 |
| TN | 700 | 57.43 | 11.57 | 4.8 | 6.87 | 19.34 |
| UT | 134 | 54.53 | 12.53 | 10.05 | 9.94 | 12.95 |
| VT | 153 | 33.98 | 36.69 | 6.66 | 9.99 | 12.68 |
| VA | 886 | 56.82 | 7.16 | 6.35 | 14.87 | 14.8 |
| WA | 1,388 | 52.06 | 10.92 | 14.43 | 8.9 | 13.7 |
| WV | 415 | 60.42 | 9.94 | 16.88 | 5.56 | 7.2 |
| WI | 807 | 59.36 | 13.22 | 8.58 | 11.48 | 7.37 |
| WY | 53 | 53.02 | 12.04 | 18.49 | 7.48 | 8.97 |

Source: Author’s calculation of the 2014-2018 American Community Survey.
